# Optimization of phytase production from potato waste using *Aspergillus ficuum*

**DOI:** 10.1007/s13205-016-0573-9

**Published:** 2016-12-02

**Authors:** Mengmeng Tian, Qiuyan Yuan

**Affiliations:** Department of Civil Engineering, University of Manitoba, Winnipeg, MB R3T 5V6 Canada

**Keywords:** Solid-state fermentation, *Aspergillus ficuum*, Phytase, Potato waste, Optimization

## Abstract

Solid-state fermentation (SSF) can divert food waste from landfills and produce high-value products. This study was aimed to investigate the feasibility of using SSF and optimize the conditions of production of phytase by *Aspergillus ficuum* from potato waste. Different parameters including pH of the potato waste, inoculum level, moisture content, incubation period, temperature, and supplementary nitrogen and carbon sources were evaluated. The results indicated that pH, inoculum level, and moisture content did not significantly vary phytase production. However, different incubation periods, incubation temperatures, nitrogen sources, and carbon sources changed the phytase production significantly. The ideal and economic conditions for phytase production consisted of a normal moisture content (79%) of potato waste, 1.0 ml inoculum size, and normal pH 6.1 at room temperature for 144 h incubation time. The highest phytase activity (5.17 ± 0.82 U/g ds) was obtained under the aforementioned optimized conditions. When (NH_4_)2SO_4_ was used as a nitrogen source in the substrate, the phytase activity increased to 12.93 ± 0.47 U/g ds, which was a 2.5-fold increase compared to the control treatment. This study proposed a novel and economical way to convert food processing waste to highly valuable products and investigated the optimal conditions of the production of phytase during SSF in potato waste.

## Introduction

Phytic acid is ubiquitous in nature and is the principal storage form of phosphorus in cereals, legumes, oilseeds, and nuts (Vohra and Satyanarayana 2003). It is known as a food inhibitor which can chelate micronutrients. Also, it is not a bioavailable source of phosphate for monogastric animals, such as human, swine, dog and cat, due to the lack of enzyme phytase in their digestive tract (Haefner et al. 2005). Therefore, extra phosphate in other digestible form has to be added into the animal feed to meet the P requirement. This will not only increase the cost of feed, but also create environmental pollution due to the surplus of P in the animal waste (Yi et al. 1996). Phytase is the primary enzyme that catalyzes the hydrolysis of phytic acid and releases inorganic phosphate, which improves the overall P digestibility by 25–30%, resulting from approximately 50% degradation of the cereal phytate (Jongbloed and Kemme 1990; Kemme et al. 1997). This will alleviate phosphorus demand in animal feed, as *P* resource is approaching depletion. Furthermore, phytase can be also applied as cosmetic additives and plant nutrition (Koshy et al. 2012; Gujar et al. 2013).

Nowadays, many types of enzymes are produced through the *Aspergillus* species (Coban et al. 2015; Kitcha and Cheirsilp 2014; Muñiz-Márquez et al. 2016; Bergstrom et al. 2016). *A. ficuum*, a filamentous fungus, is a microorganism used for phytase production (Bogar et al. 2003; Coban et al. 2015). The wide-scale industrial applications and the increasing global demand of phytase are not being met due to their high production costs. Therefore, it is necessary to produce phytase using inexpensive or renewable material as an alternative. The use of food waste from municipal solid waste (MSW) as substrate can reduce the phytase production cost by lowering the material cost.

Municipal solid waste, which is disposed in landfills, generates hazardous contaminated leachates and air emissions (EI-Fadel et al. 1997). The leachate has a significant adverse impact on public health, environment, public safety, and groundwater quality (Lu et al. 1985; Yang et al. 2008; Fatta et al. 1999; Abu-Rukah and Al-Kofahi 2001). The gaseous emission is a source of greenhouse gases (e.g., CO_2_ and CH_4_) and odors cause deterioration in the esthetic quality of the surrounding area (EI-Fadel et al. 1997). Therefore, it is important to reduce the amount of waste being sent to the landfill. Solid-state fermentation (SSF) offers a sustainable solution to divert food waste away from landfills. SSF is a fermentation process that utilizes solid substrates as support material and source of nutrients to grow microorganisms in the absence of free-flowing liquid (Pandey et al. 2000).

Food processing wastes, which are abundant in nature and rich in nutritional content, are suitable substrates for solid-state fermentation. Currently, food processing wastes such as potato waste and tofu residue are disposed by dumping in the landfill. The total production of potato worldwide was 368 million tons in 2013 as reported by the Food and Agriculture Organization of United Nation (FAO Statistic Yearbook 2013). Farming and food processing industries produced a huge amount of potato waste, which could be utilized as raw material for enzyme production. According to Mahmood et al. (1998), potato waste consists of 66.78% starch, 14.70% crude protein, 2.20% cellulose, and 3.39% pectin. The substrate used for SSF should be a nutritious material. Therefore, the high starch content makes potato waste a suitable substrate for SSF.

In solid state fermentation, the nutrient-rich organic waste can be converted to highly valuable products such as enzymes, proteins, flavors, and biologically active secondary metabolites (Raimbault 1998; Pandey et al. 2000; Pandey 2003). These products can generate substantial revenues. For example, in 2010, the technical enzymes generated $1.10 billion in revenues and the world market for industrial enzymes is expected to increase to $6 billion by 2016 (Anon 2012). The continuously growing bio-processing industry demands inexpensive and renewable material.

The end products in the SSF process are subject to the microorganism used in the fermentation process. Several studies have been carried out to investigate different microorganisms, e.g., fungi, bacteria and yeast, to produce the desirable products from various substrates. For instance, peanut press cake and rice bran produced lipases by using *Aspergillus* sp. and wheat bran produced xylanase by using *Rhizopus* sp. (Pandey et al. 2000; Behnam et al. 2016). For proper functioning of SSF processes, various operational parameters, such as moisture content and pH of substrate, and incubation temperature need to be considered, which have great impact on the effectiveness of the production rate and quality. Recently, studies on optimizing the operational parameters to increase the product yield were carried out (Kitcha and Cheirsilp 2014; Muñiz-Márquez et al. 2016; Bergstrom et al. 2016; Behnam et al. 2016). The optimal operational parameters depend on the characteristics of the microorganisms used in each process. In SSF, the moisture content has a major impact on the cell growth and enzyme production. Bogar et al. (2003) reported different phytase yields when moisture content was varied. In addition, Pal and Khanum (2010) also investigated the impact of pH on the productivity of enzymes and reported an optimum pH range (4.5–8) for enzyme production. The microbial growth is also dependent on temperature. Therefore, the use of suitable temperature is an important growth factor (Ellaiah et al. 2002).

Phytase was produced by *Penicillium purpurogenum* GE1 using corncob and corn bran under solid-state fermentation (Awad et al. 2014). Suresh and Radha (2016) showed that rice bran can produce phytase from *Rhizopus oligosporus* by solid-state fermentation. Besides, groundnut oil cake was used as substrate for phytase production by Buddhiwant et al. (2015). However, most of the previous studies on SSF were conducted on agro-industrial wastes and no research has been reported using waste from food processing as a substrate for phytase production. In this study, a new substrate, potato waste, was used to produce phytase by *A. ficuum* under SSF for the first time.

The objectives of this study were: (a) to investigate the feasibility of using the food waste fraction from food processing waste using solid-state fermentation to produce high value-added products such as phytase and (b) to optimize the conditions of phytase production using potato waste.

## Materials and methods

### Microorganisms and inoculum preparation

*Aspergillus ficuum* (ATCC^®^ 66876™) was purchased from American Type Culture Collection (ATCC, Manassas, USA). The fungus was cultured and maintained on potato dextrose agar (39 g/L PDA) slants. It was maintained by periodic transfer and stored at 4 °C. Sporulated PDA slants were used and the spores were dislodged using an inoculation needle under sterile conditions and washed with sterile DI water. The spore suspension was appropriately diluted (1 × 107 spores/ml). The inoculum was prepared by transferring 5 ml of spore suspension into a 250 ml flask containing 45 ml of sterile yeast extract peptone dextrose (50 g/L YPD) broth. The flasks were incubated on a rotary shaker at 150 rpm and 24 °C for 48 h. After 48 h of fungi cultivation, mycelial pellets were harvested and homogenized prior to inoculation.

### Substrate and characterization

The substrate was a simulated potato waste made from commercialized potato peels. The potato peels were blended to small cubes (around 0.5 × 0.5 cm) and heated for 5 min at a 100 °C water bath to reduce the particles. The resulting small soft cubes were used as substrates.

The substrate was analyzed for total solids (TS), volatile solids (VS), and total Kjeldahl nitrogen (TKN) according to the standard methods (APHA 2005). The carbon content, moisture content, and pH were also determined. The carbon content was calculated by dividing the percentage of volatile solids by 1.83 and moisture content was determined by the oven-drying method (Barrington et al. 2002). Five milliliters of DI water was added to 1 g (dry weight) of potato and the mixture was shaken for 20 min at 180 rpm at room temperature. The pH of the supernatant was determined with a pH meter (Oakton Instruments, Toronto, Canada) and it was taken as the pH value for the potato substrate.

The characteristics of the potato waste are presented in Table 1. The VS values (195.19 ± 5.33 mg/g) were close to the TS values (206.47 ± 5.16 mg/g), which indicated that organic matter composed the majority of the total solids in the potato waste. The moisture content (79.35 ± 0.52%) in potato waste was slightly lower than the average moisture content in food waste (84%) (Adhikari et al. 2008; Kiran et al. 2014; Li et al. 2008a, b; Yasin et al. 2013). The C/N ratio of potato waste was 21.96 ± 1.19, which was within the average range of C/N ratio of 20:25 found in food waste (Diaz et al. 1993).

**Table 1:** Characteristics of the potato

Characteristics	Unit	Average value
pH	–	6.13 ± 0.06
Total solids	mg/g w.w.^a^	206.47 ± 5.16
Volatile solids	mg/g w.w.^a^	195.19 ± 5.33
Moisture content	%	79.35 ± 0.52
Total carbon	% d.w.^b^	53.35 ± 0.03
TKN	mg/g d.w.^b^	24.34 ± 1.33
C/N ratio	–	21.96 ± 1.19

^a^On wet weight basis

^b^On dry weight basis

### Solid-state fermentation procedure

Solid-state fermentations tests were carried out in 500 ml Erlenmeyer flasks containing 20 g of wet potato waste in each flask. The wet substrate was sterilized at 121 °C for 15 min. After cooling down the flasks, the substrate was inoculated with mycelial suspension of the fungus. The flasks were incubated at various experimental conditions as described in the following sections. All experiments were conducted in triplicate.

SSF was carried out to study the effect of various parameters required for the optimum production of phytase by *A. ficuum* from potato waste.

### Optimization of operational parameters for phytase production

The phytase production was optimized by following ‘one variable per time’ approach to investigate different factors that affected the growth of *A. ficuum*. The influence of factors such as initial pH of the substrate (3.8, 4.7, 5.3, 6.1, and 8.2), inoculum level (0.5, 1.0, 1.5, and 2.0 ml), initial moisture content (61, 70, 77, 79, and 82%), incubation period (48, 72, 96, 120, and 144 h), and incubation temperature (22, 27, 32, 37 and 42 °C) were examined. The addition of various nitrogen sources [peptone, yeast extract, urea, NH_4_NO_3_, NH_4_Cl, and (NH_4_)_2_SO_4_] and carbon sources (glucose, lactose, sucrose, starch, and glycerol) in the substrate at 2% (w/w) concentration were also evaluated.

### Effect of different concentrations of NH_4_NO_3_ and (NH_4_)_2_SO_4_

Both NH_4_NO_3_ and (NH_4_)_2_SO_4_ showed a positive influence on phytase production in SSF. Thus, addition of NH_4_NO_3_ and (NH_4_)_2_SO_4_ to the potato waste in SSF was examined at different levels (0.2, 1, 2, and 4%).

### Enzyme extraction and phytase assay

After SSF, 25 mL DI water containing 2% CaCl_2_·2H_2_O was added to each flask. The flasks were shaken on a rotary shaker at 200 rpm for 1 h at room temperature. Then the suspension was centrifuged at 4700 rpm at 4 °C for 30 min and the clear supernatant was used as crude enzyme. It was stored at 4 °C for further preparation of the enzyme assay.

Phytase activity was determined by measuring the inorganic phosphorus released from sodium phytate solution using the method described by JECFA (JECFA 2012) with minor modifications. Sodium phytate was dissolved in an acetate buffer (0.0051 mol/L) and the final pH was adjusted to 5.50 ± 0.05. Then the buffer was mixed with crude enzyme and incubated in the water bath at 55 °C for 30 min. After incubation, the reaction was stopped by adding stop solution into the tubes. After cooling down to room temperature, absorbances were measured spectrophotometrically (BioTek^®^ Instruments, Winooski, USA) at 415 nm. The blank was the substrate inoculated with sterile DI water. The data obtained were used to calculate the activity unit of phytase (U/g ds). One phytase unit was defined as the amount of enzyme releasing 1 µmol of inorganic phosphorus per minute from 0.0051 mol/L sodium phytate under the test conditions.

### Statistical analysis

Statistical analysis was performed using SPSS (Version 18.0, Chicago, SPSS Inc.). The significance of the enzyme production with respect to different parameters was examined by one-way ANOVA, and Duncan’s multiple range test was used for multiple comparisons. Treatments were considered to have a significant effect on the result when the P value was less than 0.05 (95% confidence level). Capital letters shared in common between or among the groups indicate no significant difference.

## Results and discussion

### Effect of pH, inoculum level, and moisture content on phytase production

**Fig. 1:**
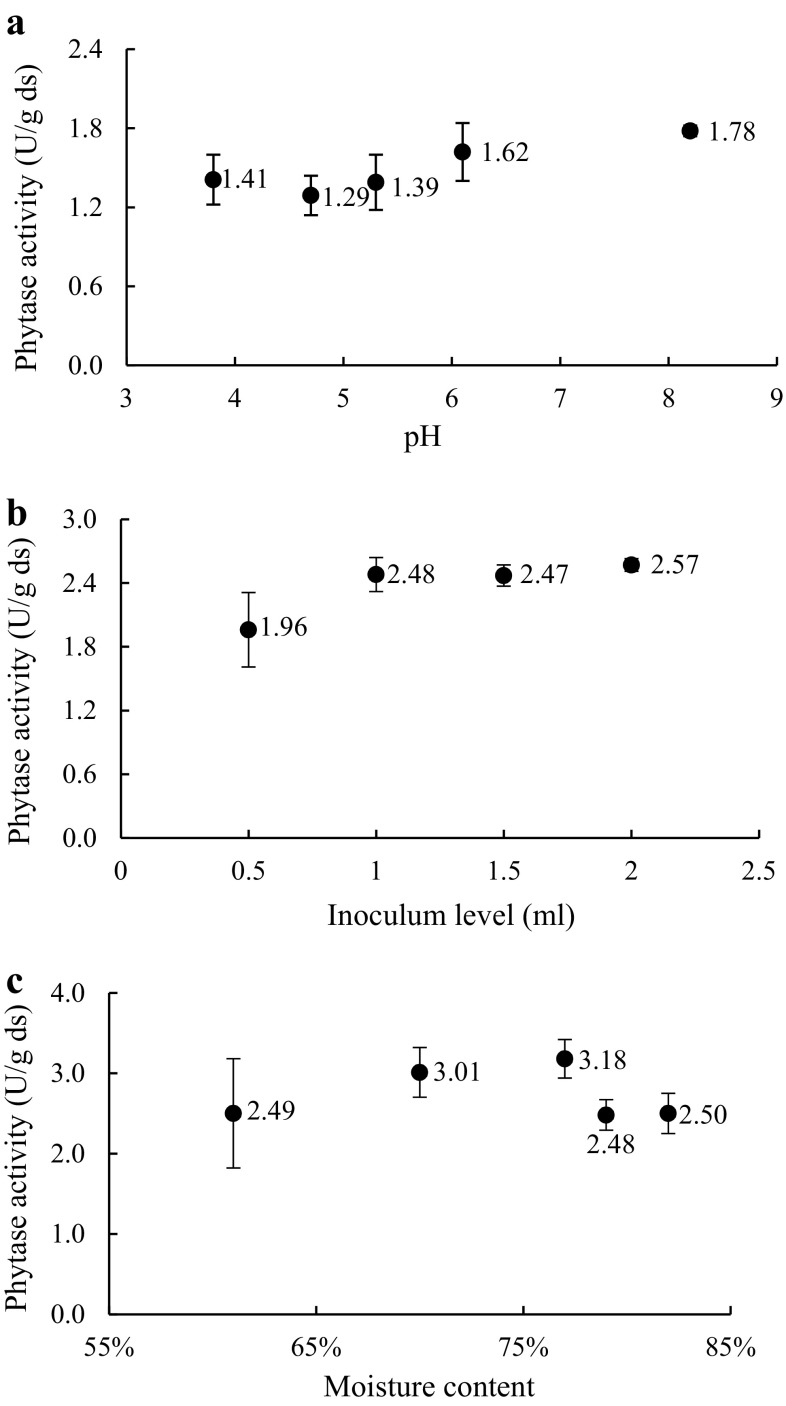
**a** Effect of initial pH on phytase production by *A. ficuum*. **b** Effect of inoculum level on phytase production by *A. ficuum*. **c** Effect of moisture content on phytase production by *A. ficuum*; *error bars* are standard deviations

The pH of the substrate, inoculum level, and moisture content are important factors that influence the production of phytase in SSF (Pandey 2003; Awad et al. 2014; Ramachandran et al. 2005). *Aspergillus* sp. showed the highest metabolic activities when the pH was around 5.0 (Muñiz-Márquez et al. 2016). The results for the range of different pH levels that were investigated in this study are shown in Fig. 1a. Generally speaking, the phytase productivity increased as the pH value increased. The maximum phytase production (1.78 ± 0.04 U/g ds) was obtained at pH of 8.2. However, phytase production for different pH conditions had no significant difference according to the one-way ANOVA results (*P* *>* 0.05). It means that pH did not have a significant influence on phytase production, which indicated that the metabolic activities of *A. ficuum* were not sensitive to the change in pH even though the maximum activity was achieved at pH 8.2. Thus, the normal pH (6.1) of the potato waste was the most economic choice and was used in the subsequent experiments.

Similarly, Awad et al. (2014) reported that *Penicillium purpurogenum* GE1 at pH 8.0 achieved the maximum phytase production, and phytase production was increased as the pH shifted to the alkaline side. However, the optimum pH for phytase production by *R. oligosporus* is 5.3 under solid-state fermentation (Sabu et al. 2002). and autoclaving changed the initial pH of the substrate to 5.3 ± 0.2 because of the buffering capacity of the substrate.

Various inoculum levels (0.5, 1.0, 1.5, and 2.0 mL) were used to study their effect on enzyme production. A similar enzyme production (2.48 ± 0.16, 2.47 ± 0.10, and 2.57 ± 0.06 U/g ds) was obtained at three higher inoculum levels (1.0, 1.5, and 2.0 mL), respectively (Fig. 1b). From one-way ANOVA, it was shown that the inoculum level had no significant influence on phytase activity (*P* *>* 0.05). The relatively high enzyme activity means better nutrients and biomass ratio at an inoculum level of 1.0 mL. A higher inoculum level also influences the moisture content of the substrate. Therefore, the inoculum level of 1.0 mL was chosen for further analyses.

The same results were also found by Vohra and Satyanarayana (2002): that inoculum size was not a significant variable for phytase production. Ramachandran et al. (2005) reported the highest enzyme production when the nutrients in the substrate and the biomass had a balanced ratio. They also found that 1.0 mL was the optimal inoculum size for phytase production by *Rhizopus* spp.

High moisture content results in substrate agglomeration and poor air content. Gautam et al. (2002) reported that the decomposition rate of the organic matter decreases at the lowest and the highest water contents. In this study, the highest enzyme activity (3.18 ± 0.24 U/g ds) was obtained when the initial moisture content was 77% (Fig. 1c) that was slightly lower than the normal potato moisture content (79.35 ± 0.52%). According to the SPSS results, there were no significant differences between phytase production at different moisture contents (*P* *>* 0.05). Therefore, the normal potato moisture content was the most economical choice.

Similar results on the effect of moisture content for phytase production were reported by Bogar et al. (2003). However, these results differed from the findings reported by Ramachandran et al. (2005) and Gautam et al. (2002). They found that the optimal moisture content was 52% by *Rhizopus* spp. and 58.3% by *A. ficuum*, respectively.

### Effect of incubation period and temperature on phytase production

**Fig. 2:**
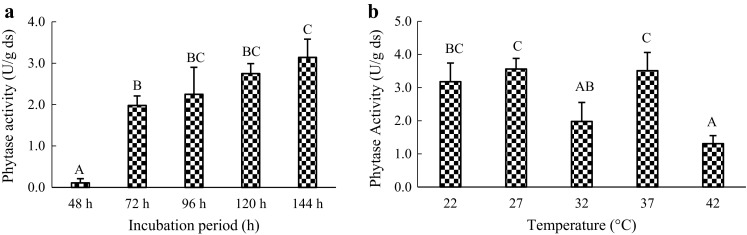
**a** Effect of incubation period on phytase production by *A. ficuum*. **b** Effect of incubation temperature on phytase production by *A. ficuum*. *Capital letters* shared in common between or among the groups indicate no significant difference according to Duncan’s multiple range test at the significance level of 0.01; *error bars* are standard deviations

The incubation period and temperature influenced the metabolic activities of fungus (Awad et al. 2014; Ramachandran et al. 2005; Wang et al. 2011). The inoculated flasks were incubated for different periods ranging from 48 to 144 h. Phytase activity in the substrate was detected every 24 h starting from 48 h. Figure 2a shows that enzyme production was increased with the growth of fungus. The maximum amount of phytase activity was observed after 144 h of incubation and the enzyme activity was 3.14 ± 0.44 U/g ds. According to the one-way ANOVA, incubation period had extremely significant influence (*P* *<* 0.01) on phytase activity. While from the results of multiple comparisons, 96, 120, and 144 h incubation period does not have significant difference, which means that longer incubation period does not result in a significant increase in phytase production. Consequently, further studies were carried out on cultures incubated for 144 h to obtain enzyme activities.

The same results were also reported by Ramachandran et al. (2005) and Wang et al. (2011). They also found that longer incubation period did not result in significant increase in enzyme production because of reduction in nutrients in the substrate. In addition, Kumari et al. (2016) showed that optimum phytase activity was obtained at 48 h incubation period by Sporotrichum thermophile with mixed substrate.

To investigate the influence of incubation temperature on phytase production, the inoculated substrates were incubated at different temperatures. The results obtained in this study indicate that the optimal temperature for maximum phytase production (3.56 ± 0.32 U/g ds) was 27 °C (Fig. 2b). Fungus grew faster at a high temperature as compared to low temperature. The high temperature also resulted in water evaporation from the substrate. Since the phytase production obtained at 22 °C did not have a significant difference with that obtained at 27 °C, the room temperature (22 °C) was used in further experiments due to economic considerations.

Awad et al. (2014) also reported that 27 °C was the optimal temperature for phytase production. The optimum temperature range for incubation of microorganisms for high phytase production is 25–37 °C (Gautam et al. 2002). However, Hussin et al. (2011) found that 33 °C was the optimum temperature for *P. stewartii* to produce phytase.

### Effect of supplementary nitrogen sources and carbon sources on phytase activity

During solid-state fermentation, all the nutrients for the microbial growth are supplied by the substrate. However, the concentration of some nutrients is insufficient in the substrate. It is necessary to provide some supplementation for these inadequate nutrients. Awad et al. (2014); Suresh and Radha (2016) reported that addition of nitrogen sources can enhance phytase production significantly. Different nitrogen sources and carbon sources (2% w/w) were investigated for their influence on enzyme production. Among various nitrogen sources, NH_4_NO_3_ produced a maximum phytase activity (9.86 ± 0.49 U/g ds) and drastically increased phytase activity (*P* *<* 0.01) (Table 2).

**Table 2:** Effect of nitrogen source on phytase production by *A. ficuum*

Nitrogen sources	Phytase activity (U/g ds)
Peptone	7.59 ± 0.26 B
Yeast extract	2.96 ± 0.42 A
Urea	7.65 ± 0.38 B
NH_4_NO_3_	9.86 ± 0.49 C
NH_4_Cl	3.61 ± 0.99 A
(NH_4_)_2_SO_4_	9.14 ± 0.81 C
Control	3.64 ± 0.54 A

Capital letters shared in common between the groups indicate no significant difference according to Duncan's multiple range test at the significance level of 0.01

Similarly, Ramachandran et al. (2005) and Suresh and Radha (2016) obtained the same results that NH_4_NO_3_ had the highest enzyme production by *Rhizopus* spp. In the case of (NH_4_)_2_SO_4_, a similar result was obtained by Bogar et al. (2003). However, Ramachandran et al. (2005) reported that (NH_4_)_2_SO_4_ inhibited phytase production. Additionally, Awad et al. (2014) reported that peptone was the most favorable nitrogen source for phytase production by *Penicillium purpurogenum* GE1. Phytase production was found to be poor in substrate containing yeast extract and NH_4_Cl. It is possible that *A. ficuum* did not utilize the nitrogen produced from yeast extract and NH_4_Cl.

Enzyme production was poor in substrate supplemented with carbohydrates. In Table 3, when compared with control, addition of glucose significantly (*P* *<* 0.01) decreased phytase production; while other carbon sources did not significantly affect phytase production.

**Table 3:** Effect of carbon sources on phytase production by *A. ficuum*

Carbon sources	Phytase activity (U/g ds)
Glucose	2.84 ± 0.53 A
Lactose	4.39 ± 0.70 B
Sucrose	3.98 ± 0.23 AB
Starch	5.08 ± 0.79 B
Glycerol	5.37 ± 0.13 B
Control	4.87 ± 0.78 B

Nevertheless, Awad et al. (2014) also found that lactose and sucrose resulted in decreased phytase production by *Penicillium purpurogenum* GE1. It is estimated that potato waste had enough carbohydrates (66.78%) for the growth of *A. ficuum* because the addition of carbon sources did not improve phytase production. On the contrary, other authors have reported that phytase production was enhanced by glucose (Wang et al. 2011; Hussin et al. 2011; Buddhiwant et al. 2015).

### Effect of addition of different concentrations of NH_4_NO_3_ and (NH_4_)_2_SO_4_ on phytase production

**Fig. 3:**
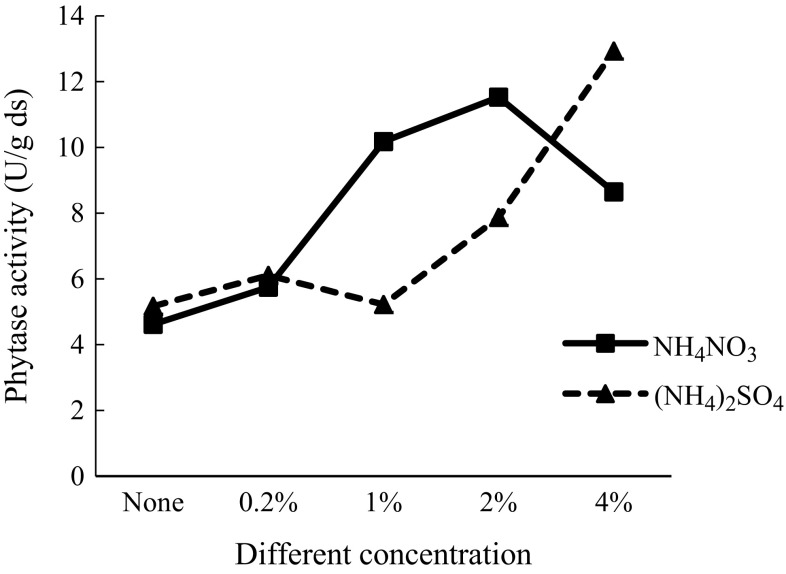
Effect of addition of different concentrations of NH_4_NO_3_ and (NH_4_)_2_SO_4_ on phytase production

As the aforementioned results showed that NH_4_NO_3_ and (NH_4_)_2_SO_4_ improved the enzyme production significantly, their optimal concentrations for enzyme yields were examined. The results are shown in Fig. 3. An addition of 2% (w/w) of NH_4_NO_3_ in potato waste was found to be the optimum concentration, as it significantly enhanced the phytase production (11.53 ± 1.11 U/g ds). The phytase production was decreased when NH_4_NO_3_ concentration was greater than 2% w/w. This indicated that the concentration of 2% w/w had a positive effect on phytase production, while a higher concentration could produce extra ammonia and cause a reduction in the biomass of the fungus. However, phytase production gradually increased as the concentration of (NH_4_)_2_SO_4_ increased to 4% w/w and reached a maximum of 12.93 ± 0.47 U/g ds, which was 2.5 times higher compared to the control that was not supplemented with (NH_4_)_2_SO_4_ (5.17 ± 0.82 U/g ds).

Ramachandran et al. (2005) reported that the lowest NH_4_NO_3_ concentration (0.5% w/w) was the most desirable for the highest phytase activity by *Rhizopus* spp. in solid-state fermentation. Li et al. (2008a, b) found that 2.3% was the best concentration of (NH_4_)_2_SO_4_ for phytase production by a marine yeast *Kodamaea ohmeri* BG3. And 0.5% (NH_4_)_2_SO_4_ resulted in the maximum phytase production by Sporotrichum thermophile (Kumari et al. 2016).

## Conclusions

Phytase production was investigated by the optimization of different parameters and supplementing different carbon and nitrogen sources. The optimization experiments showed that a maximum productivity of phytase of 5.17 ± 0.82 U/g ds were achieved by employing optimized conditions including normal moisture content of potato waste, 1.0 ml inoculum level, pH 6.1 at room temperature for incubation of 144 h. Additionally, NH_4_NO_3_ and (NH_4_)_2_SO_4_ drastically increased phytase activity as additives and 4% w/w of (NH_4_)_2_SO_4_ resulted in the highest phytase production (12.93 ± 0.47 U/g ds). Furthermore, this study showed that potato waste can be used as an economical substrate for phytase production using SSF. The microorganisms were incubated at room temperature, which made the SSF process more economical. Therefore, food processing waste can be used as a new potential substrate for enzyme production with low operational costs in phytase industry.

## References

[CR1] Abu-Rukah Y, Al-Kofahi O (2001). The assessment of the effect of landfill leachate on ground-water quality—a case study. El-Akader landfill site—north Jordan. J Arid Environ.

[CR2] Adhikari BK, Barrington S, Martinez J, King S (2008). Characterization of food waste and bulking agents for composting. Waste Manage.

[CR3] Anon (2012). Enzymes: a market survey. Focus Catal.

[CR4] APHA (2005). Standard methods for the examination of water and wastewater.

[CR5] Awad GE, Helal MM, Danial EN, Esawy MA (2014). Optimization of phytase production by Penicillium purpurogenum GE1 under solid state fermentation by using Box–Behnken design. Saudi J Biol Sci.

[CR6] Barrington S, Choiniere D, Trigui M, Knight W (2002). SE—Structures and environment: compost airflow resistance. Biosystems Eng.

[CR7] Behnam S, Karimi K, Khanahmadi M, Salimian Z (2016). Optimization of xylanase production by *Mucor indicus*, *Mucor hiemalis*, and *Rhizopus oryzae* through solid state fermentation. Biol J Microorganism.

[CR8] Bergstrom JR, Tsai TC, Kim HJ, Maxwell CV (2016). Effects of supplementation with *Aspergillus oryzae* derived phytase (Ronozyme^®^ Hiphos) and fiber degrading enzymes (Victusô^®^ Swine Starter) on growth performance, intestinal morphology, leukocyte differential, and nutrient digestibility in nursery pigs. J Animal Sci.

[CR9] Bogar B, Szakacs G, Linden JC, Pandey A, Tengerdy RP (2003). Optimization of phytase production by solid substrate fermentation. J Ind Microbiol Biotechnol.

[CR10] Buddhiwant P, Bhavsar K, Kumar VR, Khire JM (2015). Phytase production by solid state fermentation of groundnut oil cake by *Aspergillus niger*: a bioprocess optimization study for animal feedstock applications. Prep Biochem Biotechnol.

[CR11] Coban HB, Demirci A, Turhan I (2015). Microparticle-enhanced *Aspergillus ficuum* phytase production and evaluation of fungal morphology in submerged fermentation. Bioprocess Biosyst Eng.

[CR12] Diaz LF, Savage GM, Eggerth LL, Golueke CG (1993). Composting and recycling municipal solid waste.

[CR13] EI-Fadel M, Findikakis AN, Leckie JO (1997). Environmental impacts of solid waste landfilling. J Environ Manage.

[CR14] Ellaiah P, Adinarayana K, Bhavani Y, Padmaja P, Srinivasulu B (2002). Optimization of process parameters for glucoamylase production under solid state fermentation by a newly isolated *Aspergillus* species. Process Biochem.

[CR15] FAO Statistic Yearbook (2013). World food and agriculture (Statistics division).

[CR16] Fatta D, Papadopoulos A, Loizidou M (1999). A study on the landfill leachate and its impact on the groundwater quality of the greater area. Environ Geochem Health.

[CR17] Gautam P, Sabu A, Pandey A, Szakacs G, Soccol CR (2002). Microbial production of extra-cellular phytase using polystyrene as inert solid support. Bioresour Technol.

[CR18] Gujar PD, Bhavsar KP, Khire JM (2013). Effect of phytase from *Aspergillus niger* on plant growth and mineral assimilation in wheat (*Triticum aestivum* Linn.) and its potential for use as a soil amendment. J Sci Food Agric.

[CR19] Haefner S, Knietsch A, Scholten E, Braun J, Lohscheidt M, Zelder O (2005). Biotechnological production and applications of phytases. Appl Microbiol Biotechnol.

[CR20] Hussin ASM, Farouk AE, Ali AM, Greiner R (2011). Production of phytate-degrading enzyme from Malaysian soil bacteria using rice bran containing media. J Agrobiotechnol.

[CR21] JECFA (2012) Phytase from *Aspergillus niger* expressed in *A. niger*. FAO JECFA Monographs 13

[CR22] Jongbloed AW, Kemme PA (1990). Effect of pelleting mixed feeds on phytase activity and the apparent absorbability of phosphorus and calcium in pigs. Animal Feed Sci Technol.

[CR23] Kemme PA, Radcliffe JS, Jongbloed AW, Mroz Z (1997). Factors affecting phosphorus and calcium digestibility in diets for growing-finishing pigs. J Animal Sci.

[CR24] Kiran EU, Trzcinski AP, Ng WJ, Liu Y (2014). Bioconversion of food waste to energy: a review. Fuel.

[CR25] Kitcha S, Cheirsilp B (2014). Bioconversion of lignocellulosic palm byproducts into enzymes and lipid by newly isolated oleaginous fungi. Biochem Eng J.

[CR26] Koshy JC, Sharabi SE, Feldman EM, Hollier LH, Patrinely JR, Soparkar CN (2012). Effect of dietary zinc and phytase supplementation on botulinum toxin treatments. J Drugs Dermatol.

[CR27] Kumari A, Satyanarayana T, Singh B (2016). Mixed substrate fermentation for enhanced phytase production by thermophilic mould *Sporotrichum thermophile* and its application in beneficiation of poultry feed. Applied Biochem Biotechnol.

[CR28] Li SL, Kuo SC, Lin JS, Lee ZK, Wang YH, Cheng SS (2008). Process performance evaluation of intermittent-continuous stirred tank reactor for anaerobic hydrogen fermentation with kitchen waste. Int J Hydrogen Energy.

[CR29] Li XY, Liu ZQ, Chi ZM (2008). Production of phytase by a marine yeast Kodamaea ohmeri BG3 in an oats medium: optimization by response surface methodology. Bioresour Technol.

[CR30] Lu JC, Eichenberger B, Stearns RJ (1985). Leachate from municipal landfills: production and management.

[CR31] Mahmood AU, Greenman J, Scragg AH (1998). Orange and potato peel extracts: analysis and use as Bacillus substrates for the production of extracellular enzymes in continuous culture. Enzyme Microb Technol.

[CR32] Muñiz-Márquez DB, Contreras JC, Rodríguez R, Mussatto SI, Teixeira JA, Aguilar CN (2016). Enhancement of fructosyltransferase and fructooligosaccharides production by *A. oryzae* DIA-MF in solid-state fermentation using aguamiel as culture medium. Bioresour Technol.

[CR33] Pal A, Khanum F (2010). Production and extraction optimization of xylanase from *Aspergillus niger* DFR-5 through solid-state-fermentation. Bioresour Technol.

[CR34] Pandey A (2003). Solid-state fermentation. Biochem Eng J.

[CR35] Pandey A, Soccol CR, Mitchell D (2000). New developments in solid state fermentation: I-bioprocess-products. Process Biochem.

[CR36] Raimbault M (1998). General and microbiological aspects of solid substrate fermentation. Electron J Biotechnol.

[CR37] Ramachandran S, Roopesh K, Nampoothiri KM, Szakacs G, Pandey A (2005). Mixed substrate fermentation for the production of phytase by *Rhizopus* spp. using oilcakes as substrates. Process Biochem.

[CR38] Sabu A, Sarita S, Pandey A, Bogar B, Szakacs G, Soccol CR (2002). Solid-state fermentation for production of phytase by *Rhizopus oligosporus*. Appl Biochem Biotechnol.

[CR39] Suresh S, Radha KV (2016). Statistical optimization and mutagenesis for high level of phytase production *by Rhizopus oligosporus* MTCC 556 under solid state fermentation. J Environ Biol.

[CR40] Vohra A, Satyanarayana T (2002). Statistical optimization of the medium components by response surface methodology to enhance phytase production by *Pichia anomala*. Process Biochem.

[CR41] Vohra A, Satyanarayana T (2003). Phytases: microbial sources, production, purification, and potential biotechnological applications. Crit Rev Biotechnol.

[CR42] Wang ZH, Dong XF, Zhang GQ, Tong JM, Zhang Q, Xu SZ (2011). Waste vinegar residue as substrate for phytase production. Waste Manage Res.

[CR43] Yang K, Zhou XN, Yan WA, Hang DR, Steinmann P (2008). Landfills in Jiangsu province, China, and potential threats for public health: leachate appraisal and spatial analysis using geographic information system and remote sensing. Waste Manage.

[CR44] Yasin NHM, Mumtaz T, Hassan MA (2013). Food waste and food processing waste for biohydrogen production: a review. J Environ Manage.

[CR45] Yi Z, Kornegay ET, Ravindran V, Lindemann MD, Wilson JH (1996). Effectiveness of Natuphos phytase in improving the bioavailabilities of phosphorus and other nutrients in soybean meal-based semipurified diets for young pigs. J Animal Sci.

